# Dynamic and Static Functional Gradient in Temporal Lobe Epilepsy With Hippocampal Sclerosis Versus Healthy Controls

**DOI:** 10.1111/cns.70298

**Published:** 2025-04-23

**Authors:** Kangrun Wang, JiaYao Li, Fangfang Xie, Chaorong Liu, Langzi Tan, Jialinzi He, Xianghe Liu, Ge Wang, Min Zhang, Haiyun Tang, Danlei Wei, Jingwan Feng, Sha Huang, Jinxin Peng, Zhuanyi Yang, Xiaoyan Long, Bo Xiao, Juan Li, Lili Long

**Affiliations:** ^1^ Department of Neurosurgery The First Affiliated Hospital of Wenzhou Medical University, Wenzhou Medical University Wenzhou Zhejiang China; ^2^ Department of Neurology, Xiangya Hospital Central South University Changsha Hunan China; ^3^ Clinical Research Center for Epileptic Disease of Hunan Province, Xiangya Hospital Central South University Changsha Hunan China; ^4^ Department of Radiology, Xiangya Hospital Central South University Changsha Hunan China; ^5^ Department of Neurology, Zhuzhou Central Hospital Zhuzhou Hunan China; ^6^ Department of Neurosurgery, Xiangya Hospital Central South University Changsha Hunan China; ^7^ National Clinical Research Center for Geriatric Disorders, Xiangya Hospital Central South University Changsha Hunan China

**Keywords:** dynamic, fMRI, gradient, temporal lobe epilepsy

## Abstract

**Aims:**

The gradient captures the continuous transitions in connectivity, representing an intrinsic hierarchical architecture of the brain. Previous works hinted at the dynamics of the gradient but did not verify them. Cognitive impairment is a common comorbidity of temporal lobe epilepsy (TLE). Gradient techniques provide a framework that could promote the understanding of the neural correlations of cognitive decline.

**Methods:**

Thirty patients with TLE and hippocampal sclerosis and 29 matched healthy controls (HC) were investigated with verbal fluency task‐based functional MRI and gradient techniques. The correlation between task‐based activation/deactivation and healthy gradients, task‐based gradients, and dynamic features calculated with sliding window approaches was compared between HC and TLE.

**Results:**

The allegiance in the real data of HC and TLE was more widespread compared to static null models. TLE has lower dynamic recruitment of gradient, atypical activation‐gradient correlation, and contracted principal gradient. Correlation analysis proved that the reconfiguration of principal gradient did not drive the reorganization of activation. The atypical activation pattern and impaired recruitment were correlated with cognition scales in TLE.

**Discussion:**

The principal gradient is dynamic. TLE disrupted activation/deactivation patterns, the principal gradient, and the dynamics of the gradient, which were correlated with cognitive decline.

## Graphical abstract

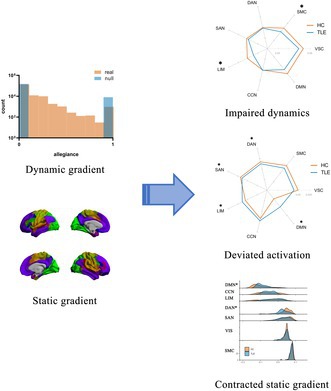

## 1 Introduction

Cognitive impairment is a common comorbidity of temporal lobe epilepsy (TLE) [1]. Task‐based functional MRI (tb‐fMRI) captured cognitive‐related activation/deactivation patterns and functional connectivity shifts in TLE. Specifically, working memory, letter verbal fluency, and naming tasks underpinned weakened activation at task‐positive functional networks and deactivation at the default mode network (DMN) [2, 3, 4] and disrupted functional connectivity between networks [5, 6]. The majority of previous studies divided brains into functional networks, such as the salience network (SAN), dorsal attention network (DAN), and DMN, based on an atlas, and considered them as separate and distinct identities. However, recent works have highlighted the continuous transition of function in the cortex and the hierarchical nature of cerebral organization.

Gradient, a flourishing continuous measure that depicts local transitions in connectivity [7, 8], was produced from functional connectivity matrices by a dimensionality reduction technique based on manifold algorithms. The principal gradient captures the hierarchical neural transition from unimodal motor/sensory areas to transmodal DMN [7]. In light of prior findings, the functional gradient is the presentation of an intrinsic hierarchical architecture of the brain in the context of functional connectivity. The principal gradient is phylogenetically conserved and in concord with geodesic distance, representing a primary dimension of cortical expansion [7, 9, 10, 11, 12]. Meanwhile, the activation/deactivation patterns during cognitive tasks complied with the principal gradient [4, 13].

Gradient techniques contributed to decomposing the neural basis of neurological disorders. The contraction of the principal gradient had been observed in autism [12], schizophrenia [13], and Rolandic epilepsy [10]. The expansion of the gradient was disrupted in Rolandic epilepsy and was correlated with cognitive decline and cortical gene expression [10]. The activation/deactivation pattern of patients with TLE deviated from the gradient of healthy controls, correlating with memory and language impairment [4, 11]. Since the principal gradient has a structural and phylogenetic basis, the resting‐state gradient was considered a static architecture, neglecting the dynamic nature of the brain. Compared to static measures, dynamic functional interactions lay the foundation of cognitive processes and presented a stronger correlation with cognition [14]. Recent studies in tb‐fMRI validated the reliability of task‐based gradients, which predicted cognitive scores with superior accuracy [15, 16, 17, 18]. Interestingly, the principal gradient contracted with cognitive load growth during the working memory [17] and semantic association [18] tasks, hinting that the task‐based principal gradient was dynamically modulated.

By performing a comprehensive analysis of the functional gradient in patients with TLE and HC, this study aims to demonstrate the dynamic nature of the functional gradient and explore the dynamic and static gradient reorganizations in patients with TLE.

## 2 Methods

### 2.1 Subjects

Thirty well‐characterized patients with TLE and hippocampal sclerosis and 29 matched healthy controls (HC) were consecutively selected from the TLE imaging database of the Xiangya Hospital (Table 1). The details of the recruitment, quality control, and clinical factors of TLE are provided in the Supporting Information and Table S1. Age, sex, years of education, overall cognitive function (Montreal Cognitive Assessment (MoCA) [19] scores and Digit Symbol Substitution Test scores), semantic verbal fluency (VFS) scores, phonemic verbal fluency (VFP) scores [20], working memory (Digit Span Test scores), visuospatial ability (Block Design Test scores), 3DT1 imaging, and fMRI of all selected participants were extracted.

**TABLE 1:** Demographic data and cognitive scales.

	HC	TLE	*p*
*N*	29	30	—
Age, y, median (IQR)	26.0 (18.0)	28.5 (9.0)	0.65
Sex, male/female	14/15	14/16	1.00
Education, y, median (IQR)	12.0 (7.0)	10.5 (7.0)	0.41
MoCA, median (IQR)	29.0 (3.0)	26.0 (5.0)	0.04^a^
VFS, median (IQR)	45.0 (29.0)	35.0 (13.0)	0.05^a^
VFP, median (IQR)	36.0 (28.0)	20.0 (17.0)	0.002^a^
DSST, median (IQR)	60.0 (24.0)	59.0 (16.0)	0.37
DST, mean (SD)	8.2 (1.6)	7.4 (1.2)	0.09

Abbreviations: SST, digit symbol substitution test; DST, digit span test; HC, healthy controls; IQR, interquartile range; MoCA, montreal cognitive assessment; SD, standard deviation; TLE, patients with temporal lobe epilepsy; VFP, verbal fluency Pinyin test; VFS, semantic verbal fluency test.

^a^ FDR‐corrected.

The neuroimages were collected at the MRI center of Xiangya Hospital with a Siemens MAGNETOM Prisma 3.0 T MR scanner. The 3DT1 imaging was collected with the magnetization‐prepared rapid acquisition with gradient echo sequence (field of view 233 mm, repetition time 2.11 s, echo time 3.18 ms, flip angle 9°, 320 × 320 matrix). The fMRI was collected with the gradient echo planar T2‐weighted sequence (field of view 225 mm, repetition time 1 s, echo time 37 ms, flip angle 52°, 90 × 90 matrix). Participants performed the covert VFC task [20] during fMRI scanning.

The VFC task contains five blocks. Each block contains a 30 s rest module and a 30 s task module, in order. In rest modules, participants should stare at the black fixation presented on a white background and rest. During task modules, a black Chinese character would be presented on a white background. Participants should covertly generate Chinese words that start with the given Chinese character as much as possible. During the whole task, participants are required to remain silent to minimize head motion. Before the scan, participants finished an out‐of‐scan verbal fluency scale identical to the VFC task but with different Chinese characters. The scores of the verbal fluency scale are the numbers of non‐repetitive compliant words generated and were used to estimate in‐the‐scan performance.

Written informed consent was obtained from all participants. The study was approved by the ethics committee of Xiangya Hospital of Central South University.

### 2.2 Imaging Preprocessing

Imaging data were preprocessed with the Statistical Parametric Mapping 12 (available at https://www.fil.ion.ucl.ac.uk/spm/). The preprocessing pipeline includes realignment, coregistration, segmentation, normalization to the Montreal Neurological Institute space, and spatial smoothing (6 mm). Preprocessed images were used for activation analysis performed by SPM12. For gradient analysis, images were further preprocessed by toolbox CONN v.20.b [21]. A bandpass filter (0.009–0.10 Hz) removed high‐and low‐frequency noises. Head motion, outlying scans, modular effects, and signals inside white matter and cerebral spinal fluid were regressed out.

### 2.3 Dynamic and Static Gradient

With toolbox CONN v.20.b, averaged task‐based functional time series in each parcel of Schaefer parcellation [22] (400 ROI scale) were extracted and were used to calculate 400 × 400 functional connectivity matrices. Static functional connectivity gradients were then computed following a previously reported approach [7, 8]. Matrices were binarized into unweighted matrices with 10% connectivity density. The averaged resting‐state functional connectivity matrix of healthy controls was generated from resting‐state fMRI data from the Human Connectome Project (HCP) database by Vos de Wael R. et al. [23] Toolbox BrainSpace [8] was used to calculate the principal task‐based functional connectivity gradient with the recommended setting: approach = diffusion embedding, kernel = normalized angle, sparsity = 0.9.

For dynamic gradient, the 300 s task was divided into 14 windows of 40 s, in steps of 20 s [14]. The functional connectivity matrices were calculated for each window, forming 14 consecutive matrices for each participant or group. The window‐specific individual‐level gradients were generated with the aforementioned method. Each gradient was discretized into 20 equally sized bins along the gradient [16, 24].

### 2.4 Gradient‐Based Measures

According to the Yeo network parcellation [25], 400 ROIs were distributed to the somatomotor cortex (SMC), visual cortex (VIS), SAN, DAN, (para)limbic system (LIM), cognitive control network (CCN), and DMN. Since the ROIs are different in size, the measures of a network were defined as the voxel‐number‐weighted mean measures of the ROIs distributed to this network.

To quantify the dynamic organization of gradients, the allegiance matrix of the gradient of HC was calculated. Allegiance is the probability that node i and node j are assigned to the same bin in the set of gradients constructed from all HC and all windows [26]. The allegiance matrices of HC and the static null models were compared with Kolmogorov–Smirnov tests [14].

The flexibility [27] and recruitment [26] were used to characterize the dynamic features of gradients. Flexibility is the probability that a ROI was assigned to different bins in two contiguous windows. Recruitment of a ROI is the possibility that this ROI and other ROIs from the same network are divided into the same bin throughout 14 windows.

The averaged beta signals (task > rest) were extracted from each ROI and each subject using RESTplus v1.27 [28]. A permutation test was used to compare the correlation between a healthy gradient and the activation/deactivation pattern of HC and TLE. The loading scores [11] were calculated to evaluate the network‐specific task‐related reorganization in patients. The loading scores were the averaged beta signals weighted by the corresponding resting‐state gradient. A higher loading score indicated that the activation profile was more conformed to the gradient. The mean loading scores of seven networks [25] were compared between HC, TLE‐HS, and TLE‐NHS. The resting‐state gradient was recommended for loading score calculation [11] because it ensured the independence between beta signals and the gradient to avoid autocorrelation.

The individual‐level matrices were averaged to group‐level matrices of patients and HC, which were then used to generate group‐level gradients. The distribution of the gradient of each network was compared between groups [4, 25].

### 2.5 Statistical Analysis for Demographic and Clinical Data

Statistical analysis was conducted by R studio (available at https://www.rstudio.com/) and IBM SPSS Statistics 23 (available at https://www.ibm.com/products/spss‐statistics). The normality of data was tested using the Shapiro–Wilk test. Analysis of covariates (ANCOVA), or Quade nonparametric ANCOVA, and post hoc pairwise comparisons, Mann–Whitney *U* test, Chi‐square test, or Fisher exact test was applied when applicable. Pearson correlation between gradient‐based statistics and neuropsychological scales or clinical features (disease duration and seizure frequency) was calculated. Age, sex, years of education, and MoCA were controlled as covariates.

Alpha level was set at *p* < 0.05 with appropriate FDR‐correction.

## 3 Results

### 3.1 Construction and Validation of Functional Connectivity Gradient

**FIGURE 1:**
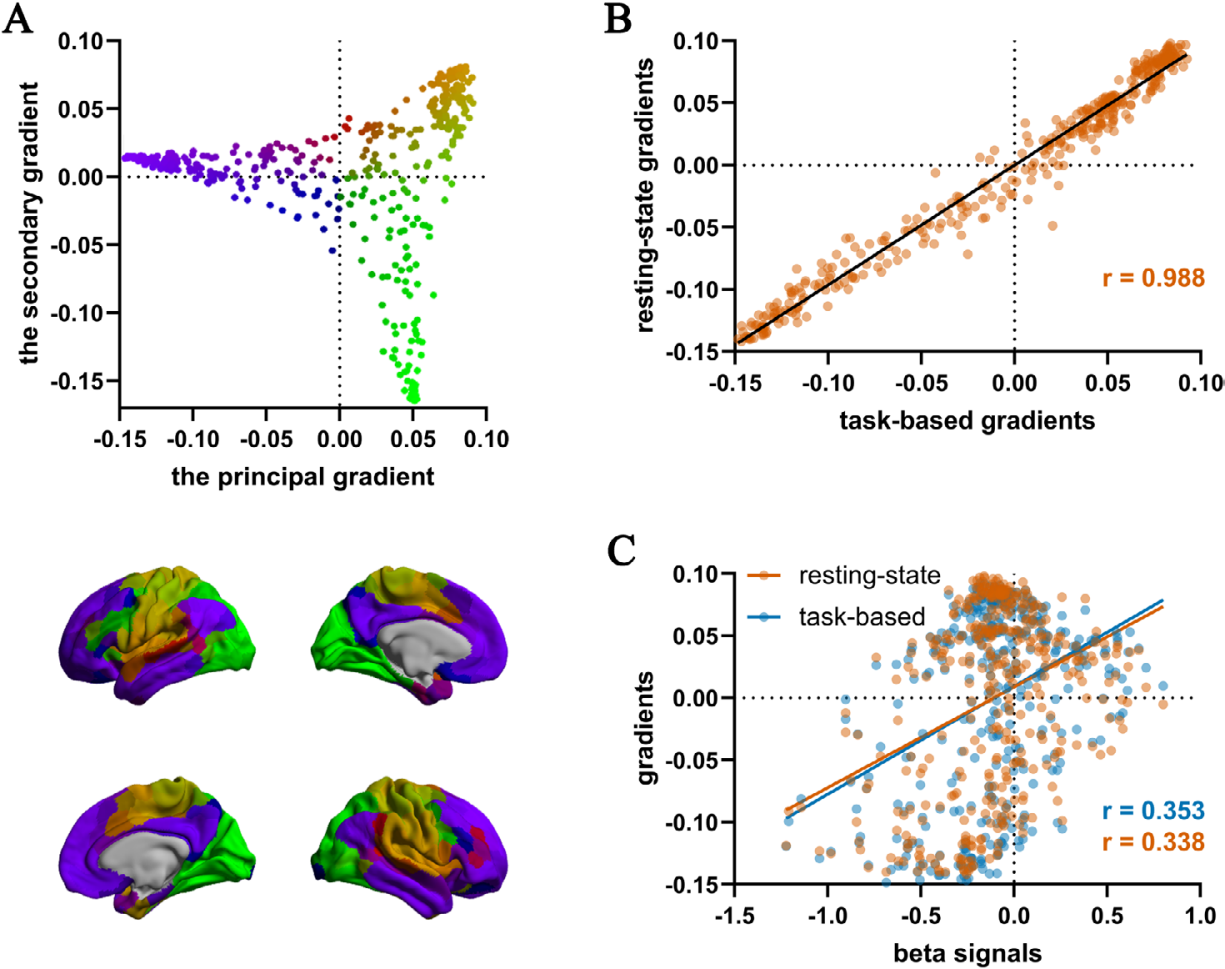
Validation of gradient techniques in task‐based data. (A) The task‐based gradient in healthy controls; (B) correlation between resting‐state gradient and task‐based gradient; (C) correlation between beta signals and the principal gradients.

First, the functional connectivity gradient approaches were validated in the current cohort. The task‐based functional connectivity gradient of the Chinese population virtually mirrored the resting‐state functional connectivity gradient of HCP healthy controls (Figure 1A). The principal gradient captured the functional shift from unimodal to transmodal regions. The second gradient is anchored at one end by visual regions and at the other end by sensorimotor regions [7].

The Chinese‐population‐derived task‐based gradient was correlated with the HCP database‐derived resting‐state gradient (*p*_unc_ < 0.001, Figure 1B). In addition, the beta signals in HC were correlated with the resting‐state gradient and the task‐based gradient (*p*_unc_ < 0.001), indicating that the healthy activation/deactivation pattern followed the functional gradients (Figure 1C). These results suggested that the functional connectivity gradient was consistent in task‐based and resting‐state data, as well as in the Chinese population and the HCP database.

### 3.2 Dynamics of Functional Gradient

**FIGURE 2:**
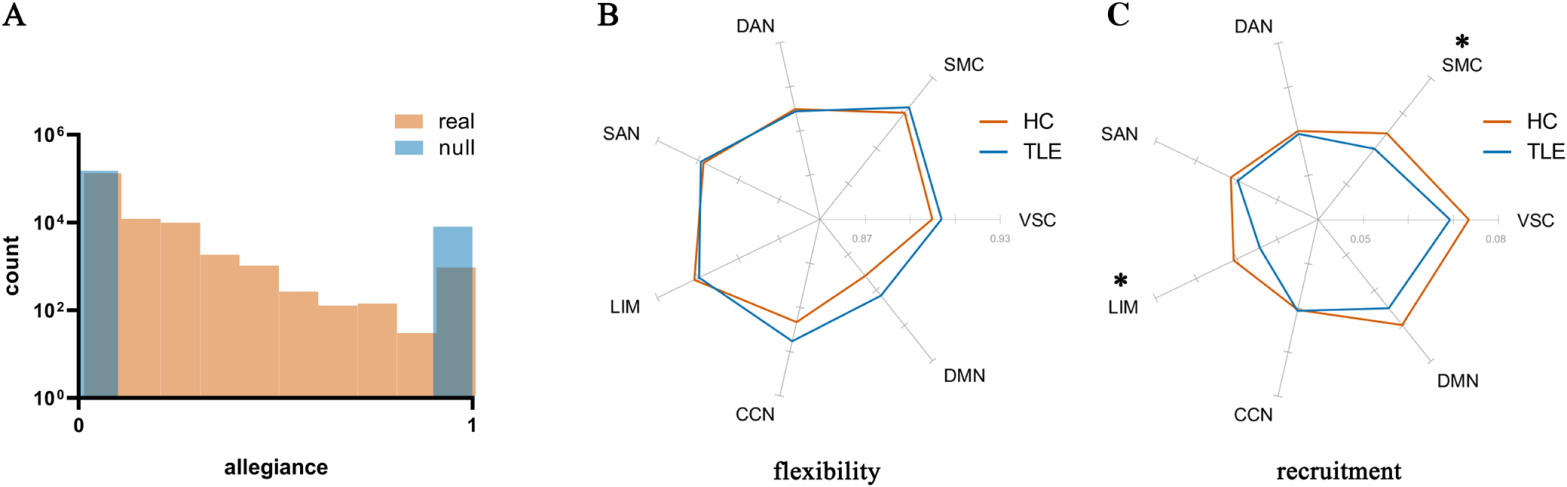
Dynamic gradient. (A) the distribution of allegiance in real data and the static null model; (B) the flexibility of networks; (C) the recruitment of networks. CCN, cognitive control network; DAN, dorsal attention network; DMN, default mode network; HC, healthy controls; LIM, Limbic system; SAN, salience network; SMC, somatomotor cortex; TLE, temporal lobe epilepsy; VIS, visual cortex; *, *p* < 0.05.

Having confirmed the availability of gradient‐based processes in the current cohort, the dynamic nature of the functional connectivity gradient is demonstrated by comparing the real data with static null models. The allegiance in the real data of HC and TLE was more widespread compared to static null models (*p*_unc_ < 0.001, Figure 2A), proving the dynamic modulation of the gradient during the task.

Two widely tested characteristics of dynamic networks, flexibility and recruitment, were compared between patients and controls. The recruitment of SMC (*p*_unc_ = 0.04) and LIM (*p*_FDR_ = 0.03) was decreased in TLE (Figure 2B), indicating lower functional consistency of SMC and LIM.

### 3.3 Task‐Related Reorganization

**FIGURE 3:**
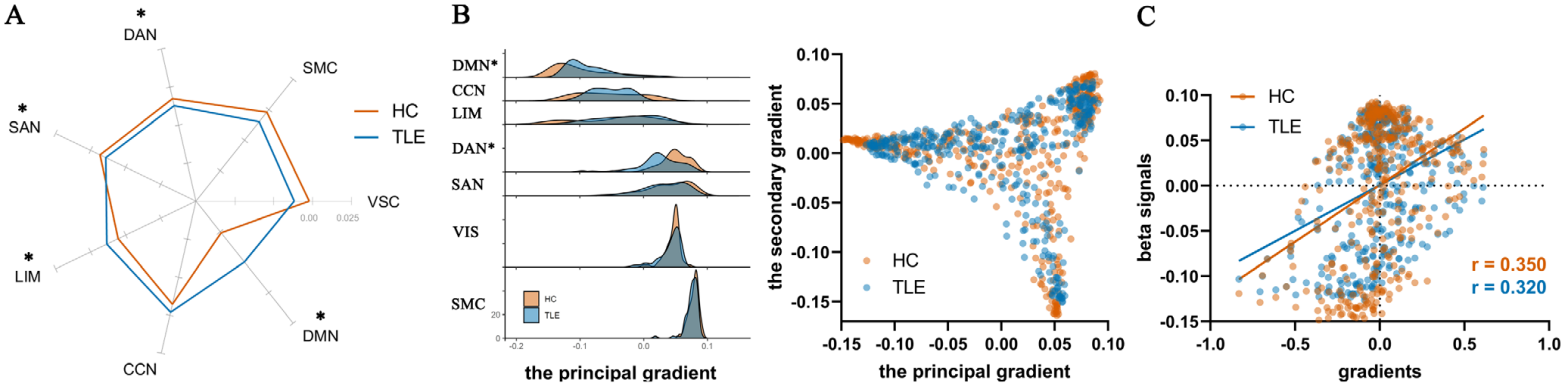
Static gradient. (A) the loading scores of networks; (B) the distribution of the gradients in HC and TLE; (C) the correlation between beta signals of TLE and the principal gradient of HC or TLE. CCN, cognitive control network; DAN, dorsal attention network; DMN, default mode network; HC, healthy controls; LIM, Limbic system; SAN, salience network; SMC, somatomotor cortex; TLE, temporal lobe epilepsy; VIS, visual cortex; *, *p* < 0.05.

Next, the previously reported inconsistency between activation/deactivation patterns and the main gradient [4] was reproduced with the current cohort. Though the correlation between the healthy resting‐state gradient and the activation/deactivation pattern did not differ between HC and patients (permutation test, *p*_unc_ = 0.77), TLE presented atypical activation‐gradient relationships (Figure 3A) in the DAN (*p*_unc_ = 0.05), SAN (*p*_unc_ = 0.04), LIM (*p*_unc_ = 0.02) and DMN (*p*_unc_ = 0.04). Specifically, LIM and DMN in TLE deviated from the healthy gradient compared to HC, while SAN and DAN, on the contrary, were more compliant with the gradient in TLE than in HC. Since the healthy activation pattern was correlated with the main gradient, the gradient reorganization in TLE was explored. The gradient of DMN was compressed (*p*_FDR_ = 0.002), and the DAN was expanded (*p*_FDR_ = 0.002) in TLE (Figure 3B).

To ascertain whether the activation reorganization is driven by gradient reconfiguration, we compared the correlation between activation patterns of patients and the gradient of patients or HC with a permutation test. The activation patterns of patients with TLE were more correlated with the gradient of HC than the gradient of patients with TLE (*p*_unc_ = 0.02, Figure 3C), suggesting that the task‐related reorganization did not result from gradient reconfiguration.

### 3.4 Clinical and Cognitive Correlation

Then came the last research question: whether the gradient features contributed to functional decline in TLE. VFP scores were positively correlated with the recruitment of SMC (*p*_unc_ = 0.03) and LIM (*p*_FDR_ = 0.004) and loading scores of DMN (*p*_FDR_ = 0.05, FDR‐corrected). Digit Span Test scores were positively correlated with the recruitment of SMC (*p*_unc_ = 0.04). Higher Digit Symbol Substitution Test scores were correlated with lower loading scores of DAN (*p*_FDR_ = 0.05) and higher loading scores of DMN (*p*_FDR_ = 0.05).

### 3.5 Sensitivity Analysis

Several sensitivity analyses were conducted to rule out potential confounders.

- The number of bins might influence the outcomes of dynamic measures [14]. Hence, the dynamic features were recalculated with the number of bins set at 7, the same as the number of networks. Lower recruitments of SMC (*p*_unc_ = 0.04) and LIM (*p*_unc_ = 0.02) were still observed in TLE.
- Task‐based gradient of HC was used to calculate loading scores instead of resting‐state gradient. There was no significant difference between the correlation between healthy task‐based gradient and activation/deactivation of HC and that of patients (permutation test, *p*_unc_ = 0.90). The loading scores of LIM (*p*_unc_ = 0.02) and DMN (*p*_unc_ = 0.03) were lower in patients compared to HC.
- Loading scores, gradients, flexibility, and recruitment were compared between HC, left, and right TLE to address the lateralization effect. In short, the loading scores and gradients were shifted both in the left and right TLE. The recruitment was lower in the right TLE but not in the left TLE. Details were provided in Supporting Information and Figure S1.
- Antiseizure medications (ASMs) could influence the activation/deactivation pattern [29] and functional connectivity [30]. There was no significant Pearson correlation between the number of ASMs and gradient measures (*p*_unc_ > 0.1).

## 4 Conclusion

Dynamic and hierarchy have been recognized as fundamental characteristics of brain architecture. With a combination of dynamic and gradient analyses in tb‐fMRI, the present study provided a perspective of dynamic and static gradients in neurotypical controls and typical cognition‐defective populations (i.e., TLE). Task‐based gradients were essentially dynamic, while the networks of TLE less congregated dynamically and correlated with cognitive impairment.

This study first tried to address the dynamics of the gradient in HC and TLE and confirmed that the principal gradient was indeed dynamic during the task by comparing verbal fluency task data with a static null model. The principal gradient had been observed to change with different cognitive demand [17, 18]. These results further demonstrated that the gradient was flexible throughout the task, not just when conditions changed. The controls‐patients comparison revealed that the recruitment, a measure of intra‐network dynamic communication [26], was reduced in the SMC and LIM of TLE. Recruitment is the probability that a node would be assigned to the same community with other nodes from the same functional network, representing the functional stability and modular organization of certain networks. A stable modular organization of networks balances the cognitive requirement of information local processing and global transferring [31]. Reduced recruitment of executive, language, and salience networks has been observed in internet gaming disorder [32], temporal lobe epilepsy [14], and major depressive disorder [33] respectively, consistent and correlated with certain cognitive impairments in certain disorders. During cognitive tasks, the recruitment of networks represents a response to higher cognitive loads [17, 34]. In the current study, the recruitment reduction was associated with disease duration. With the epileptogenic focus of TLE in LIM and the terminal of epileptic discharges in SMC [35], LIM and SMC may endure the most severe destruction in epilepsy. Patients with TLE failed to upregulate the recruitment of SMC and LIM in response to higher semantic and cognitive demands for concentrating and searching unusual words [18], resulting in worse working memory and verbal fluency.

The abnormal activation/deactivation pattern had been widely observed in TLE [2, 4, 5]. Recent studies revealed the atypical activation/deactivation pattern in TLE [4, 11], verified by the current study. Though counterintuitive, the correlation between the activation/deactivation pattern and a healthy gradient could be stronger in TLE than in HC [11]. The task‐related effect could be influenced by different cognitive demands and types of tasks used [4, 11]. The deflection of LIM and DMN activation/deactivation patterns represents TLE‐related functional disruptions [2, 36]. Moreover, the compliance shown in SAN and DAN potentially reflects a futile or even detrimental attempt at compensation, as the correlation analysis proved.

Consistent with works in psychological disorders [12, 13] and Rolandic epilepsy [10], this study found a contracted principal gradient in TLE. There are major differences in clinical presentation between psychological and epileptic disorders: autism spectrum disorder is characterized by fixed interests, repetitive behaviors, and communication disorder [37]; schizophrenia is characterized by delusions, hallucinations, thought disorganization, alogia, withdrawal, and blunt [38]; Rolandic epilepsy is characterized by age‐dependent nocturnal convulsive seizures [10]; and temporal lobe epilepsy is characterized by aura followed by focal impaired awareness seizures. Despite different manifestations, the clinical phenotype of these disorders could be interpreted by a dysfunction developed from low‐level (sensorimotor) to high‐level function (cognition), and they share several rare risk genes and have high comorbidities with each other [39, 40]. Maldevelopment of the cortex, as demonstrated by gradient‐based studies, is a shared etiology of psychological and epileptic disorders. The principal gradient this study observed in TLE is similar to the gradient reported in Rolandic epilepsy, characterized by compressed DMN and expanded DAN [10], potentially representing a shared hierarchical defect in epilepsy. Interestingly, the activation/deactivation pattern of TLE showed a stronger correlation with the gradient of HC, compared to the gradient of TLE, indicating a decoupling between the activation/deactivation pattern and gradient, and the activation/deactivation deviation was not driven by gradient reconfiguration. Though the principal gradient is functional connectivity‐based, it is associated with spatial geometry [7] and gene expression [10] of cortex, potentially representing a basic architecture that was more resistant to functional connectivity reorganization. Since the gradient of healthy controls represents the efficient activation/deactivation distribution, the compensatory reorganization of functional connectivity may pull the activation/deactivation pattern toward the healthy gradient in TLE. As a result, the gradients of networks were not correlated with cognitive performance, while the activation/deactivation pattern and dynamics of gradient were highly associated with verbal fluency and overall cognition, as observed. These results further suggest that the static gradient depicts a basic architecture derived from gene and development, while the dynamics of gradients and activation/deactivation patterns represent flexible functional impairment and compensation of neuropsychological disorders. The demonstration and exploration of dynamic gradient in HC and TLE have provided us with a tool to further understand the neural basis of normal and deficient cognition. Dynamic gradient measures could serve as biomarkers and indicators for cognitive impairment in the clinical practice of TLE.

The strengths of the current study include well‐characterized participants, robust methodology, comprehensive mapping of static and dynamic gradient measures, and several sensitivity analyses. This study has limitations. First, patients with TLE had significantly worse overall cognitive function. MoCA scores were used as covariates of no interest in group comparison to rule out the effect of overall cognitive function. However, it has to be acknowledged that this might dilute the group difference. Second, the VFC task is covert. Such design avoids excessive head motion but also impedes real‐time monitoring of performance. The out‐of‐scanner verbal fluency scales were used to estimate in‐the‐scanner performance, and participants who were considered to have not actively participated in the task during the scan were excluded (no significant activation cluster in the left frontal lobe). Third, ASMs have an impact on activation/deactivation patterns and functional connectivity [27, 28]. Though the number of ASMs was not correlated with gradient measures, specific ASMs could still influence fMRI results. While levetiracetam was reported to restore normal fMRI signals, some ASMs, such as topiramate and zonisamide, have a severe impact on activation/deactivation and functional connectivity [28, 41]. Though none of the patients were taking topiramate or zonisamide, ASMs with mild to moderate impact are administered. Future studies with larger sample sizes are encouraged to unscramble the effect of different types and combinations of ASMs. Fourth, this is a cross‐sectional study without longitudinal data. Future follow‐up studies could provide more information on how the dynamic and static gradient disruptions evolve with disease progression. Fifth, we conducted a pooled analysis for left and right TLE due to a limited sample size. Due to the asymmetry of the language network, the functional network alteration differs between left and right TLE [6]. To explore the lateralization effect, we conducted separate analyses for the left and right TLE in the sensitivity analysis. The results of activation/deactivation and static gradient roughly replicated those of the main analysis. Meanwhile, the reduction of recruitment is more prominent in the right TLE, according to previous reports [6, 42]. The contralateral functional connectivity and functional variability are increased in the right TLE, leading to increased inter‐network connection and decreased recruitment, potentially representing an adaptive or destructive reaction to seizures, which is detrimental to cognition.

In conclusion, the present study provides evidence for the dynamic nature of the functional gradient, verifies atypical activation/deactivation patterns, and functional gradients in patients with TLE. The activation/deactivation patterns and dynamic gradients were associated with cognitive decline. This work fills a knowledge gap in the features of the principal gradient, promotes a comprehensive understanding of the neural correlation of functional impairment in TLE, and provides targets for treatments of cognitive decline.

## Ethics Statement

The study was approved by the ethics committee of Xiangya Hospital of Central South University (201803420).

## Consent

Written informed consent was obtained from all participants.

## Conflicts of Interest

The authors declare no conflicts of interest.

## Supporting information


Data S1.

## Data Availability

The data and codes that support the findings of this study are available at https://osf.io/fzcwy/.
