# Supplementary material for: Dynamic and Static Functional Gradient in Temporal Lobe Epilepsy With Hippocampal Sclerosis Versus Healthy Controls
Source: CNS Neurosci Ther. 2025 Apr 23;31(4):e70298. doi: 10.1111/cns.70298 (PMC12015638; doi:10.1111/cns.70298)
Supplement: Supplementary file 1 — Data S1. [file CNS-31-e70298-s001.docx]

**Dynamic and static functional gradient in temporal lobe epilepsy**

*-- ONLINE SUPPLEMENT --*

**Contents:**

1. the details of epilepsy imaging database of the Xiangya Hospital

2. lateralization effect in TLE

3. Figure S1

4. Table S1

**1. the details of epilepsy imaging database of the Xiangya Hospital**

The epilepsy imagine database of the Xiangya Hospital contains multimodal MRI, clinical characteristics, EEG, neuropsychological scales, and demographic characteristics of patients with epilepsy and matched healthy controls since December 2018. The inclusion criteria are (i) right-handed native Chinese; (ii) no history of neurological or psychiatric disease except for epilepsy; (iii) able to comprehend our language paradigm and sign the written informed consent; (iv) definitive diagnosis according to semiology, clinical history, electroencephalography, and MRI by two certified neurologist (L.L. and B.X.) based on the definition of International League Against Epilepsy (ILAE)^1^.

Hippocampal sclerosis was diagnosed with following criteria: (i) decreased hippocampal volume, increased temporal horn volume, gray–white matter boundary blurring, asymmetrical hippocampus, loss of internal structure, or increased T2 signal^2^, visually evaluated by two experienced neuroimagers (F.X. and H.T.); (ii) smaller than the 95% reference hippocampus volume, calculated by Hipposeg^3^. A blind rater (L.L.) reconciled the disagreement between the neuroimagers and Hipposeg^4^.

Participants will finish a neuropsychological scale battery and then receive MRI scanning. Functional MRI data was preprocessed and used to calculate the individual-level beta maps and head motion parameters. Quality control excluded: (i) excessive head motion (maximum head motion/rotation over 3mm/degree or sudden motion/rotation over 1.5mm/degree that cannot be regressed out) in 2/0 HC/TLE; (ii) gross artefact in 0/1 HC/TLE; (iii) lack of participation during the task (lack of activation in left frontal lobe under p_unc_ < 0.01) in 0/0 HC/TLE; and (iv) insufficient field of view in 0/1 HC/TLE.

**2. lateralization effect in TLE**

We compared the gradient-based parameters between HC, left TLE, and right TLE (Figure S1). The loading scores of LIM (left TLE vs. HC, p_FDR_ = 0.006; right TLE vs. HC, p_unc_ = 0.03) and DMN (left TLE vs. HC, p_unc_ = 0.04; right TLE vs. HC, p_FDR_ = 0.04) decreased in both left and right TLE. Compared to HC, the gradient of DAN (left TLE vs. HC, p_FDR_ = 0.003; right TLE vs. HC, p_FDR_ = 0.003) was expanded and the gradient of DMN (left TLE vs. HC, p_FDR_ < 0.001; right TLE vs. HC, p_FDR_ < 0.001) was contracted in both left and right TLE. The recruitment of SMC (p_FDR_ = 0.003) and DMN decreased in the right TLE (p_FDR_ = 0.002) compared to HC. As for parameters that did not present significant intergroup difference in the main analysis, the gradient of VIS was expanded in right TLE compared to HC (p_FDR_ < 0.001) and left TLE (p_FDR_ < 0.001). The gradient of SMC was shifted to transmodal end in left TLE compared to HC (p_FDR_ < 0.001) and right TLE (p_FDR_ < 0.001). The gradient of SAN was shifted to transmodal end in left TLE compared to HC (p_FDR_ = 0.02).


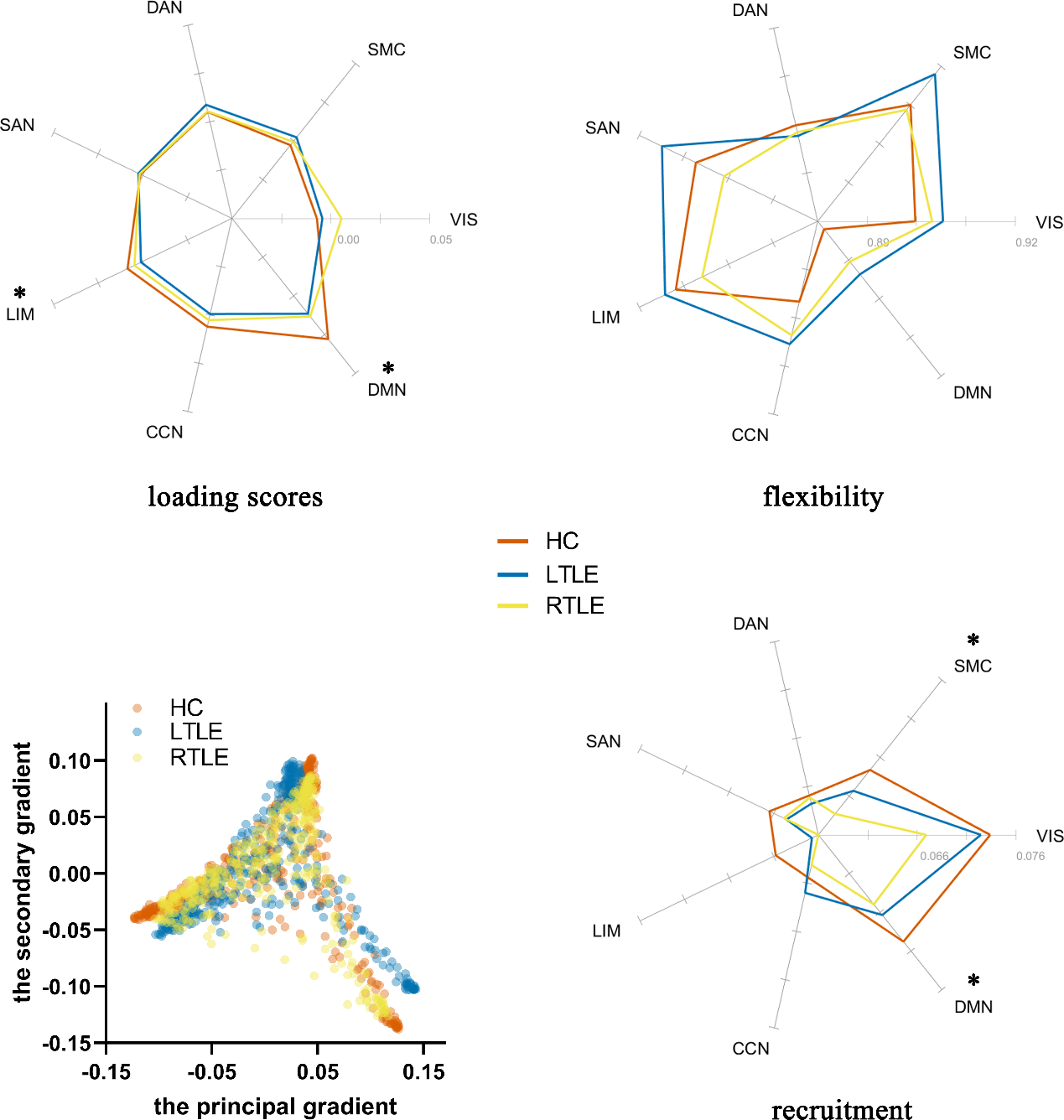


Figure S1. Comparison between HC, LTLE, and RTLE

CCN, cognitive control network; DAN, dorsal attention network; DMN, default mode network; HC, healthy controls; LIM, Limbic system; LTLE, left temporal lobe epilepsy; RTLE, right temporal lobe epilepsy; SAN, salience network; SMC, somatomotor cortex; VIS, visual cortex; *, p < 0.05.

| Clinical factors | TLE |
| --- | --- |
| Number of ASMs |  |
| 1 | 16 |
| 2 | 13 |
| 3 | 1 |
| AOO, mean (SD) | 17.8 (7.6) |
| Duration, median (IQR) | 9.5 (14.0) |
| Seizure frequency |  |
| Daily | 7 |
| Weekly | 13 |
| Monthly | 6 |
| Yearly | 4 |
| History of FBTCS | 9 (30.0%) |

Table S1. Clinical factors

AOO, age of onset; FBTCS, ASM, antiseizure medications; focal-to-bilateral tonic-clonic seizures; IQR, interquartile range; SD, standard deviation; TLE, patients with temporal lobe epilepsy.

1. Fisher RS, Acevedo C, Arzimanoglou A, et al. ILAE official report: a practical clinical definition of epilepsy. *Epilepsia*. Apr 2014;55(4):475-82. doi:10.1111/epi.12550

2. Mo J, Liu Z. Automated detection of hippocampal sclerosis using clinically empirical and radiomics features. *Epilepsia*. Dec 2019;60(12):2519-2529. doi:10.1111/epi.16392

3. Winston GP, Cardoso MJ, Williams EJ, et al. Automated hippocampal segmentation in patients with epilepsy: available free online. *Epilepsia*. Dec 2013;54(12):2166-73. doi:10.1111/epi.12408

4. Cook MJ, Fish DR, Shorvon SD, Straughan K, Stevens JM. Hippocampal volumetric and morphometric studies in frontal and temporal lobe epilepsy. *Brain*. Aug 1992;115 ( Pt 4):1001-15. doi:10.1093/brain/115.4.1001
